# Mysteries of α1-antitrypsin deficiency: emerging therapeutic strategies for a challenging disease

**DOI:** 10.1242/dmm.014092

**Published:** 2014-04

**Authors:** Raafe Ghouse, Andrew Chu, Yan Wang, David H. Perlmutter

**Affiliations:** 1 Department of Pediatrics, University of Pittsburgh School of Medicine, One Children’s Hospital Drive, 4401 Penn Avenue, Pittsburgh, PA 15224, USA.; 2 Children’s Hospital of Pittsburgh of UPMC, One Children’s Hospital Drive, 4401 Penn Avenue, Pittsburgh, PA 15224, USA.; 3 Department of Cell Biology, University of Pittsburgh School of Medicine, 3500 Terrace Street, 5362 Biomedical Sciences Tower, Pittsburgh, PA 15261, USA.

**Keywords:** α1-antitrypsin deficiency, Autophagy, Liver disease

## Abstract

The classical form of α1-antitrypsin deficiency (ATD) is an autosomal co-dominant disorder that affects ~1 in 3000 live births and is an important genetic cause of lung and liver disease. The protein affected, α1-antitrypsin (AT), is predominantly derived from the liver and has the function of inhibiting neutrophil elastase and several other destructive neutrophil proteinases. The genetic defect is a point mutation that leads to misfolding of the mutant protein, which is referred to as α1-antitrypsin Z (ATZ). Because of its misfolding, ATZ is unable to efficiently traverse the secretory pathway. Accumulation of ATZ in the endoplasmic reticulum of liver cells has a gain-of-function proteotoxic effect on the liver, resulting in fibrosis, cirrhosis and/or hepatocellular carcinoma in some individuals. Moreover, because of reduced secretion, there is a lack of anti-proteinase activity in the lung, which allows neutrophil proteases to destroy the connective tissue matrix and cause chronic obstructive pulmonary disease (COPD) by loss of function. Wide variation in the incidence and severity of liver and lung disease among individuals with ATD has made this disease one of the most challenging of the rare genetic disorders to diagnose and treat. Other than cigarette smoking, which worsens COPD in ATD, genetic and environmental modifiers that determine this phenotypic variability are unknown. A limited number of therapeutic strategies are currently available, and liver transplantation is the only treatment for severe liver disease. Although replacement therapy with purified AT corrects the loss of anti-proteinase function, COPD progresses in a substantial number of individuals with ATD and some undergo lung transplantation. Nevertheless, advances in understanding the variability in clinical phenotype and in developing novel therapeutic concepts is beginning to address the major clinical challenges of this mysterious disorder.

## Introduction

**Fig. 1.**
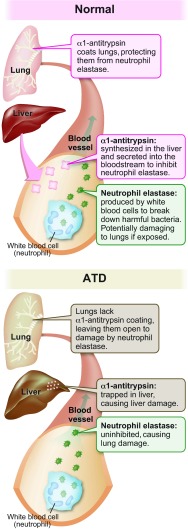
Pathophysiology of α1-antitrypsin and its deficiency (ATD). Synthesis, secretion and function of AT in the circulation and lungs of an unaffected individual (top) and illustration of how the liver and lungs are adversely affected in the classical form of ATD (bottom) (image used with permission from The University of Utah Genetic Science Learning Center, http://Learn.Genetics.utah.edu).

α1-antitrypsin deficiency (ATD) was first described 50 years ago when absence of the α1-globulin fraction was noticed in the serum specimens of individuals with chronic obstructive pulmonary disease (COPD; Box 1 provides a glossary of clinical terms) (Laurell and Eriksson, 1963). Later, this deficiency was found in an infant with cirrhosis (Sharp et al., 1969), establishing a link between ATD and liver disease; however, the mechanism by which ATD could lead to liver disease remained one of its most perplexing characteristics for many years and raised further questions about how the deficiency of an enzyme inhibitor could cause different diseases in two organ systems. Several breakthroughs led to the recognition that lung disease is caused predominantly by a loss-of-function mechanism whereas liver disease involves a gain-of-toxic-function mechanism. One of the breakthroughs was the realization that the major function of α1-antitrypsin (AT) is inhibition of neutrophil elastase and, when circulating levels of AT are reduced, this serine proteinase can destroy the connective tissue matrix of the lung, causing emphysema (Silverman and Sandhaus, 2009). This conceptual advance led to the protease-antiprotease imbalance paradigm that constitutes the molecular basis for COPD; i.e. excess protease and/or deficiency of antiprotease predisposes individuals to this disease. Another series of discoveries led to the recognition that ATD is characterized by a point mutation that renders AT, a hepatic secretory glycoprotein, prone to misfolding and accumulation in the early portions of the secretory pathway, specifically the endoplasmic reticulum (ER) of liver cells (reviewed in Perlmutter, 2011). Studies in transgenic mice have provided evidence that intracellular accumulation of the mutant misfolded protein, named α1-antitrypsin Z (ATZ), is proteotoxic. In the liver, the primary site of AT synthesis, ATZ accumulation causes liver fibrosis and carcinogenesis by a gain-of-function mechanism (Hidvegi et al., 2010). Together, these breakthroughs have shown that loss of antiprotease function is the dominant mechanism for lung disease and accumulation (proteotoxicity) of ATZ in hepatocytes is the primary mechanism for liver disease (Fig. 1).

Another perplexing aspect of ATD that has emerged over the years is the marked variation in clinical severity of lung and liver disease among affected homozygotes. The classical form of ATD is an autosomal co-dominant disorder that affects 1 in 3000 live births in many populations; however, a significant number of homozygotes escape both liver and lung disease. There is also a marked variation in the age of onset and severity of lung disease (Silverman and Sandhaus, 2009). We now know that one environmental modifier, cigarette smoking, markedly increases the severity of lung disease and that other genetic and environmental modifiers are also involved. Analysis of a unique cohort of homozygotes identified by an unbiased newborn screening study carried out in Sweden has shown that only ~8% of homozygotes develop clinically significant liver disease in the first 4 decades of life (Piitulainen et al., 2005; Sveger, 1976), indicating that genetic and/or environmental modifiers play a major role in whether a homozygote falls into the subpopulation that is susceptible to liver disease or is somehow protected from liver disease. This variation in clinical phenotype has added to the challenge of detection and diagnosis of ATD and means that therapeutic strategies have to be tested in and designed for specific subgroups of the affected, already rare, population. In this review, we will discuss advances in our understanding of liver and lung disease of ATD, pathogenic mechanisms, and novel therapies that are now being investigated to treat this clinically challenging disease.

Box 1. **Clinical terms**

Ascites: pathologic accumulation of fluid in the peritoneal cavity
Augmentation therapy: treatment involving intravenous infusion of pooled α1-antitrypsin collected from the plasma of healthy donors
Biliary atresia: end result of a destructive, idiopathic, inflammatory process that affects intrahepatic and extrahepatic bile ducts, leading to fibrosis and obliteration of the biliary tract and eventual development of biliary cirrhosis
Bronchiectasis: abnormal, chronic enlargement of the bronchi
Cholestasis: physiologically defined as a measurable decrease in bile flow and pathologically defined as the histological presence of bile pigment in hepatocytes and bile ducts
Chronic obstructive pulmonary disease (COPD): preventable and treatable disease characterized by airflow limitation that is not fully reversible. The airflow limitation is usually progressive and is associated with an abnormal inflammatory response of the lungs to noxious particles or gases, primarily caused by cigarette smoking. Although COPD affects the lungs, it produces significant systemic consequences. Manifestations of COPD range from dyspnea, poor exercise tolerance, chronic cough with or without sputum production and wheezing to respiratory failure
Cirrhosis: diffuse liver process characterized by fibrosis and the conversion of normal liver architecture into structurally abnormal nodules. Cirrhosis represents a dynamic state reflecting the competing processes of cell injury (necrosis), response to injury (fibrosis) and regeneration (nodule formation)
Dyspnea: shortness of breath
Ecchymoses: subcutaneous extravasation of blood causing discoloration of the skin
Emphysema: pathological term for destruction of the gas-exchanging surfaces of the lung
FEV_1_: also known as forced expiratory volume; a measure of the maximum amount of air that can be expelled in the first second during a forced vital capacity (FVC) determination
Fibrosis: the formation of excess fibrous connective tissue (scar tissue) in an organ
FVC: forced vital capacity; the volume delivered during an expiration made as forcefully and completely as possible starting from full inspiration
Hepatocellular carcinoma: malignant tumor that arises from the hepatocytes
Huntington’s disease: neurodegenerative genetic disorder that affects muscle coordination and leads to cognitive decline and neuropsychiatric manifestations
Hypercholesterolemia: the presence of high levels of cholesterol in the blood
Hypersplenism: disorder involving enlarged spleen size and premature sequestration of circulating blood cells in the spleen, often resulting in abnormal decreases in circulating levels of white blood cells, red blood cells and/or platelets; associated with a variety of disorders, including cirrhosis due to chronic liver disease (see also ‘Splenomegaly’ and ‘Cirrhosis’)
Jaundice: the visible yellow discoloration of soft tissues (skin, eyes) due to accumulation of bile pigments in these tissues; a common manifestation of chronic liver disease
Lucency: in radiology, a region in an image caused by an absorber of lower X-ray attenuation than its surrounding tissues
Portal hypertension: clinical syndrome hemodynamically defined as an increase in the pressure gradient across the liver (between portal pressure and inferior vena cava pressure) above the normal value of 5 mmHg
Pruritus: sensation of intense, persistent itching; a common clinical manifestation of chronic liver disease
Splenomegaly: enlargement of the spleen, as detected by clinical examination or radiological measurement of spleen size (see also ‘Hypersplenism’)

## Clinical characteristics of ATD-associated liver disease

How is liver disease that is caused by ATD clinically recognized? Forms of this liver disease manifest in infancy, childhood, adolescence or later in adult life. The usual presentation in infants, persistent jaundice, is noted at 1–2 months of age. These infants often have elevated serum conjugated bilirubin and transaminase levels, suggesting liver involvement. In some cases there might also be an enlarged liver. The infantile presentation of ATD can closely resemble that of extrahepatic biliary atresia in terms of overall clinical appearance and laboratory evaluation. The clinical presentation can also be characterized by pruritus and hypercholesterolemia, and it is important to distinguish this from inborn errors of bile secretion that cause infantile cholestasis. More severe liver dysfunction reflecting portal hypertension, such as gastrointestinal bleeding, ascites, splenomegaly and hypersplenism, and poor growth, is occasionally seen in cases of ATD in infancy (reviewed in Perlmutter, 2011).

Liver disease caused by ATD that comes to clinical attention during childhood or adolescence is almost always recognized because of the effects of portal hypertension. In adults, it is recognized either because of the sequelae of portal hypertension or because of hepatocellular carcinoma (see Case study box). This reflects the pathological effects of mutant ATZ accumulation in the liver, leading to fibrosis and/or cirrhosis, and hyperproliferation of hepatocytes. An autopsy study carried out in Sweden showed a highly significant association of ATD and hepatocellular carcinoma, well beyond what might be associated with cirrhosis alone (Eriksson et al., 1986). Over the past 20 years there seems to have been an increase in the prevalence of the ‘adult’ form of ATD liver disease. Indeed, over the last 10 years, 85–90% of all liver transplant procedures performed in the United States for ATD were for adults, typically at around 50 to 65 years of age (United Network of Organ Sharing website, http://optn.transplant.hrsa.gov/). A significant number of adult patients with ATD who come to clinical attention have other causes of liver disease, including alcoholic liver disease, non-alcoholic steatohepatitis, or features of hemochromatosis or autoimmune liver disease.

Much of what is known about the prevalence of liver disease in ATD and its outcome comes from a prospective, relatively unbiased study started in Sweden in the 1970s (Piitulainen et al., 2005; Sveger, 1976). In this nationwide screening study, 200,000 newborns were screened and 127 homozygotes identified. From this group of homozygotes, 14 had prolonged obstructive jaundice and 9 of the 14 had severe liver disease. A little more than 50% of the remaining infants had elevated blood transaminase levels but no other signs of liver disease. This cohort has been followed relatively completely over the ensuing 40 years, and we know that only ~8% of the population has developed clinically significant liver disease. One of the limitations of this cohort study is that liver biopsies were not performed, so many homozygotes could have smoldering hepatic fibrosis that will first present clinically as the ‘adult’ form of the disease in the sixth or seventh decade of life.

It is still very difficult to prospectively predict which individuals with ATD will develop severe liver disease. Treatment is directed at alleviating the complications of portal hypertension but liver transplantation is the only therapeutic strategy currently available for severe liver disease caused by ATD.

## Clinical characteristics of lung disease in ATD

What are the clinical effects of ATD on the lungs? ATD predisposes individuals to premature development of COPD with panacinar emphysema. It is characterized by progressive decline in forced expiratory volume (FEV_1_). Dyspnea is the most common symptom, and many patients report cough, phlegm production and wheezing, either chronically or with acute viral upper respiratory tract infections (Janus et al., 1985). The long-term loss of pulmonary functional capacity correlates with the incidence of respiratory infections (Dowson et al., 2001). COPD associated with ATD often shows a characteristic radiographic pattern with hyperinflation and marked lucency at the lung bases (see Case study box). In one study of individuals with ATD, emphysematous abnormalities were most prominent at the bases in 64% of patients and at the apices in 36% (Parr et al., 2004).

**Case study**

A 52-year-old male presents for evaluation of progressive fatigue and easy bruising. His history is notable for a chronic intermittent cough and several episodes of pneumonia in the last few years, which were treated with outpatient antibiotics. He has experienced increased shortness of breath with physical exertion. He uses albuterol inhalers as needed during intermittent respiratory symptoms and, despite counseling, he continues to smoke one pack of cigarettes daily. Family history reveals that his father died from complications of pulmonary disease that was attributed to heavy tobacco use. He also had a sibling twin who died in infancy of liver failure of unknown etiology. On physical examination, he has scattered ecchymoses along his extremities, diffuse wheezing and crackles throughout his lung fields, a firm liver edge 2 inches below the costal margin, and a palpable spleen. Laboratory assessments reveal mild elevations in his liver transaminases (ALT=67 IU/l, AST=52 IU/l) with decreased albumin (2.9 g/dl) and elevated INR (international normalized ratio for prothrombin time) (1.8). Chest X-ray shows hyperinflated, hyperlucent lung fields. Liver ultrasound reveals diffusely coarsened echotexture suggestive of fibrosis as well as a 2 cm focal hypoechoic lesion in the right hepatic lobe. α1-antitrypsin phenotype returns as PiZZ with a decreased serum level of AT of 31 mg/dl.

This case presents several of the challenges found in ATD. Without a high degree of suspicion for ATD, clinicians might interpret pulmonary abnormalities as evidence of other more common entities like asthma. Liver disease can follow an insidious course, with transaminases often being normal or minimally elevated, making them an unreliable indicator of liver injury. Significant fibrosis and hepatocellular carcinoma might not be detected until liver pathology is advanced. Even with early detection, there are currently no therapies for ATD-associated liver disease beyond transplantation. For lung disease, augmentation therapy is available, but long-term efficacy is questionable. Another interesting aspect of this case is the sibling twin who died of liver disease, which could have been a severe infantile presentation of ATD-associated liver disease. Marked clinical variability is a hallmark of ATD and is probably a function of genetic modifiers such as polymorphisms in genes that regulate autophagy.

ATD has also been associated with bronchiectasis. An early study indicated that bronchiectasis was present in 11.3% of 246 individuals with ATD (Larsson, 1978). The estimated prevalence in other reports has varied widely, from 2% to 43% (Piras et al., 2013; Cuvelier et al., 2000). The exact relationship between bronchiectasis and ATD remains a mystery. ATD should be considered in the differential diagnosis of bronchiectasis along with cystic fibrosis, primary immunodeficiency, allergic bronchopulmonary aspergillosis and disorders that affect ciliary function, such as primary ciliary dyskinesia.

Lung disease does not affect ATD individuals during childhood. However, the onset of emphysema in ATD individuals can be earlier than in non-ATD individuals, who usually present in the sixth and seventh decades of life. The National Heart, Lung, and Blood Institute-sponsored registry for patients with ATD found the mean age to be 46 years (Alpha1-Antitrypsin Deficiency Registry Study Group, 1994). Multiple studies have demonstrated that ATD is a strong risk factor for early-onset COPD, but not every ATD individual is destined to develop COPD. Risk factors include chronic bronchitis, frequent pneumonias, family history of COPD, and cigarette smoking. Cigarette smoking increases the risk of developing fixed airflow obstruction and can markedly accelerate the onset of dyspnea by as much as 19 years, leading to earlier and more severe pulmonary disease. The effect of cigarette smoking is thought to be due to oxidative and proteolytic inactivation of residual AT function by active oxygen intermediates and metalloproteases released from mononuclear phagocytes (Crystal, 1990; Stockley et al., 2009). The rate of decline in FEV_1_ is four times greater in smoking than in nonsmoking persons with ATD (Janus et al., 1985).

Increased susceptibility to COPD in ATD is predominantly due to a loss-of-function mechanism. In normal individuals, AT bathes the elastin-rich extracellular matrix in the interstitium of the lung parenchyma, protecting them from neutrophil elastase. Homozygotes for ATD are susceptible to premature development of emphysema because reduced AT activity in the lungs permits uninhibited proteolytic damage to their connective tissue matrix. However, there is substantial variability in the development of disease among individuals who are homozygous for the Z allele even when cigarette smoking is taken into consideration (DeMeo et al., 2009). Genome-wide association studies and integrative genomic approaches in COPD have demonstrated significant associations in ATD patients for single-nucleotide polymorphisms (SNPs) in the chromosome 15q region that includes *CHRNA3* (cholinergic nicotine receptor alpha3) and *IREB2* (iron regulatory binding protein 2) (Kim et al., 2012). Other potential modifiers of lung disease in ATD include *NOS3*, *GSTP1*, *TNF* and *IL10*.

Most individuals with lung disease due to ATD are being treated with purified AT replacement therapy. Although this treatment corrects the anti-elastase deficiency, there is still limited evidence of clinical efficacy (Dickens and Lomas, 2011). This could be because this therapy is only instituted clinically at a stage in which the emphysematous process is irreversible or, alternatively, that other mechanisms contribute to the lung disease pathogenesis. There is also recent evidence that pharmacological protocols for AT replacement therapy have anti-inflammatory and immunomodulatory effects that are independent of its elastase-inhibitory properties (Jonigk et al., 2013). Each year, a number of patients with ATD lung disease, often severely affected at an early age, thereby undergo lung transplantation (Tanash et al., 2011).

## Cellular mechanisms that determine liver disease

How does the liver get damaged in ATD? It is now well established that the mechanism of liver disease in ATD involves gain-of-toxic-function mechanisms. The strongest evidence comes from the outcome of expressing the mutant *ATZ* gene in transgenic animals. The so-called PiZ mouse model of ATD was generated by using as a transgene a genomic fragment that encodes all exons and introns of human *ATZ*, including ~2 kb of upstream and downstream flanking regions (Sifers et al., 1987; Carlson et al., 1989). In addition to the intrahepatocytic globular inclusions that are the histological hallmark of the disease, these mice develop hepatic fibrosis and hepatocellular carcinoma that closely phenocopies the liver in humans with ATD (Hidvegi et al., 2010; Marcus et al., 2010). Recently, a *Caenorhabditis elegans* model of ATD was reported to show reduced longevity, delayed development and decreased brood size, providing further validation for a proteotoxic effect of ATZ accumulation in a living organism (Gosai et al., 2010).

**Fig. 2.**
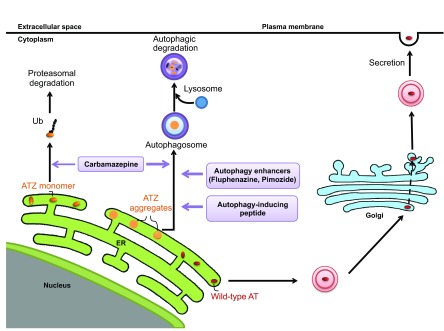
Pathways for the disposal of the mutant form of AT, ATZ, as potential therapeutic targets in ATD. Unlike wild-type AT, which efficiently traverses the conventional secretory pathway, the majority of mutant ATZ accumulates in the ER. Soluble forms of ATZ (ATZ monomers) are presumably degraded by the proteasomal pathway, whereas insoluble forms of ATZ (ATZ aggregates) are degraded by autophagy. Emerging candidate therapies that target these disposal pathways include carbamazepine, which has been shown to increase both proteosomal and autophagic disposal of ATZ. Autophagy enhancers such as certain phenothiazines and the novel Tat-beclin 1 peptide, an autophagy-inducing peptide, enhance autophagic disposal of ATZ. Ub, ubiquitin.

Although the molecular details of the process by which intracellular accumulation of mutant ATZ elicits proteotoxic effects are not known, cellular responses to ATZ accumulation have been characterized in model systems to determine which intracellular degradation pathways participate in the disposal of the mutant protein and the signaling pathways that are activated, presumably to facilitate cellular adaptation. The results of these studies have led to the recognition that the proteasomal and autophagic degradation pathways play a major role in disposal of ATZ (Fig. 2). The role of the proteasomal pathway in ATZ disposal has been demonstrated in yeast and mammalian systems, and involves a process named ER-associated degradation (ERAD), in which substrates for degradation in the ER are delivered to the proteasome in the cytoplasm by retrograde translocation (Brodsky, 2012; Qu et al., 1996; Werner et al., 1996).

Autophagy also plays a major role in the disposal of accumulated ATZ. Autophagy is a ubiquitous pathway by which cells digest internal constituents to generate amino acids as a mechanism for homeostatic turnover and to survive stresses such as starvation. It is characterized by the formation of double-membrane vacuoles in the cytoplasm, which sequester cytoplasm and subcellular organelles and then fuse with lysosomes, with consequent degradation of the internal constituents. The autophagic pathway has been shown to play a crucial role in the degradation of aggregated proteins, and defects in autophagic function, including the known decline in autophagy with aging, have been implicated in the pathogenesis of degenerative diseases (Rubinsztein et al., 2011). Autophagy was first implicated in ATD when abundant autophagosomes were observed in mammalian cell lines, in the PiZ mouse model and then in the liver of patients with ATD (Teckman and Perlmutter, 2000). Definitive evidence for the role of autophagy in the disposal of ATZ was provided by studies showing delayed degradation of ATZ in mammalian cell line and yeast models genetically engineered for deficits in autophagy (Kamimoto et al., 2006; Kruse et al., 2006). Studies in a novel mouse model of ATD engineered for liver-specific inducible expression of ATZ and green fluorescent autophagosomes showed that the accumulation of ATZ is sufficient to activate autophagy, suggesting that autophagy is particularly important in ATD because it is specifically activated when ATZ accumulates, and then plays a role in its disposal (Kamimoto et al., 2006). In yeast rendered autophagy-deficient by a mutation in ATG6 (beclin 1 in mammals), ATZ disposal is only impaired when levels of expression are high (Kruse et al., 2006). These results suggest that the ERAD/proteasomal pathway can handle ATZ at lower levels of expression, presumably because it is capable of degrading soluble forms of ATZ, but at higher levels of expression, as ATZ accumulates in polymers and insoluble aggregates, autophagy becomes critical for ATZ degradation. A recent study showed that the proteasomal and autophagic pathways play important roles in ATZ disposal in a live, whole-organism model of ATD in *C. elegans* (Gosai et al., 2010).

Recently, a pathway from Golgi to lysosome that is mediated by sortilin has been shown to contribute to intracellular degradation of ATZ in yeast and mammalian cell line models (Gelling et al., 2012). This pathway probably participates in the disposal of any ATZ molecules that reach the Golgi or re-cycle into the ER-Golgi intermediate compartment. Polymorphic variation in sortilin has been implicated in risk for cardiovascular disease and, based on its contribution to intracellular degradation of ATZ, it is also a candidate modifier of ATD-associated liver disease. We suspect that there are still other mechanisms by which cells degrade ATZ that have not yet been identified.

Several signaling pathways are specifically activated in response to intracellular ATZ accumulation and presumably reflect protective adaptations or what have been called ‘proteostasis’ mechanisms. Cell line and mouse models with inducible expression of ATZ have been particularly valuable for identification of these pathways because these types of systems are less prone to cellular adaptations that potentially occur in systems with constitutive expression of the mutant protein. As mentioned above, a mouse model with hepatocyte-specific inducible expression of ATZ was used to show that the autophagic response is activated when ATZ accumulates in the liver, providing evidence for the importance of autophagy as a proteostatic response to hepatic ATZ accumulation (Kamimoto et al., 2006). Similar approaches have shown that NFκB and TGFβ signaling are also a part of the distinct adaptive response of cells to ATZ accumulation (Hidvegi et al., 2005; Hidvegi et al., 2007). Because one of the downstream targets of NFκB is the transcription factor Egr1, which is essential for hepatocyte proliferation in the regenerative response to partial hepatectomy (Liao et al., 2004), we believe that NFκB activation is important in the effect of ATZ accumulation on liver cell proliferation and carcinogenesis in ATD (Hidvegi et al., 2007). Because it has been widely implicated in hepatic fibrosis (Wynn, 2007), TGFβ signaling is likely to be one of the mechanisms by which hepatic fibrosis develops in ATD. Intracellular accumulation of ATZ has minimal or no activation of the unfolded protein response (UPR) in most systems, or it leads to relatively limited activation of the UPR (Hidvegi et al., 2005). This could be due to the tendency of ATZ to polymerize rather than ‘unfold’, supported by the finding that UPR signaling is more robust when nonpolymerogenic AT variants accumulate in the ER in comparable cell and mouse systems (Hidvegi et al., 2007). The relative lack of UPR signaling when ATZ accumulates in the ER compared with when nonpolymerogenic AT variants accumulate could provide an explanation for why there is relatively limited apoptosis in the liver in ATD. However, the relationship between ATZ accumulation and UPR signaling is not well understood and awaits further in-depth investigation.

Studies by Rudnick et al. that investigated liver cell proliferation in the PiZ mouse model of ATD have shed some light on the predilection for primary liver cancer in ATD (Rudnick and Perlmutter, 2005; Rudnick et al., 2004). Liver cells that have accumulated more polymerized ATZ (so-called globule-containing hepatocytes) are relatively impaired in proliferation but induce chronic hyperproliferation in the globule-devoid hepatocytes by a trans-effect. Interestingly, most hepatocellular carcinomas described in individuals with ATD are negative for AT staining but in many cases are surrounded by AT-positive, globule-containing hepatocytes, consistent with the concept that globule-containing hepatocytes drive the chronic hyper-proliferative process that leads to carcinoma (Hadzic et al., 2006; Zhou and Fischer, 1998; Zhou et al., 2000).

Presumably, clinically significant liver disease evolves when ATZ accumulation and the resulting proteotoxicity overwhelms these cell-protective proteostatic mechanisms. According to this conceptual paradigm, genetic and environmental modifiers are likely to influence this delicate balance of proteotoxic and proteostatic forces. This idea has been validated to a certain extent by early work that demonstrated a lag in intracellular degradation of ATZ in cell lines from ATD patients with severe liver disease (Wu et al., 1994). It is also supported by the observation that drugs that enhance autophagy reduce the hepatic load of ATZ in the PiZ mouse model (Hidvegi et al., 2010). A polymorphism in ER mannosidase I, an enzyme involved in the ERAD pathway, has recently been implicated as a genetic modifier predisposing to infantile disease (Pan et al., 2009). This putative modifier mechanism is also consistent with the conceptual model that disease occurs when proteotoxicity overwhelms proteostasis mechanisms.

## Autophagy enhancer drugs for proteotoxicity

Recently, we investigated the possibility that intracellular disposal pathways could be used as targets for mitigating the ATZ accumulation/proteotoxicity that causes liver disease in ATD. We selected the autophagy pathway as a target for several reasons: it is specifically activated when ATZ accumulates in cells and in the liver *in vivo* (Kamimoto et al., 2006); it participates in the disposal of ATZ and is specialized for insoluble polymers or aggregates of ATZ (Kruse et al., 2006); and a number of drugs that stimulate autophagy have been shown to reduce the cellular load of aggregation-prone proteins that cause neurodegenerative diseases, particularly Huntington’s disease (Sarkar et al., 2007; Zhang et al., 2007). From the list of available autophagy enhancer drugs, we focused on carbamazepine (CBZ), an anticonvulsant and mood stabilizer, because it has been used so extensively in clinical practice. The results showed that CBZ could indeed mediate increased intracellular degradation of ATZ. Even more importantly, when administered orally to PiZ mice the drug mediated a reduction in hepatic ATZ load and in hepatic fibrosis (Hidvegi et al., 2010). Because CBZ is approved by the FDA for use in clinical practice, it has been moved into a Phase II/III clinical trial for patients with severe liver disease due to ATD. The results of this study also validate the concept that endogenous proteostasis mechanisms, mechanisms that probably protect ATD patients from hepatic proteotoxicity and disease, can be targeted for effective drug therapy.

By adapting a novel *C. elegans* model of ATD as a high-content drug-screening platform, we have identified several other drugs that enhance the autophagic disposal of ATZ and reduce proteotoxicity (Gosai et al., 2010). To generate the *C. elegans* model, a chimeric GFP-ATZ plasmid was used as a transgene and targeted for expression in the intestine because the intestinal cells of *C. elegans* carry out many of the functions attributed to the liver in higher organisms. The model recapitulates the cellular defect of ATD with intracellular accumulation of GFP-ATZ and proteotoxicity. Because the degree of ATZ accumulation correlates with intensity of GFP+ inclusions in the worm intestine and these fluorescent signals can be quantified, the model permits high-content analysis using automated array scanning and large-particle flow cytometry. As proof of principle, an initial screen of the Library of Pharmacologically Active Compounds (LOPAC) identified five hit compounds that induced significant, dose-dependent reductions in worm ATZ load. Of these, four compounds are known to enhance autophagic activity and are already in clinical use, providing the possibility that drug re-purposing can accelerate clinical trials for treatment of ATD-associated liver disease. Furthermore, two of the compounds are from the phenothiazine family, a drug family that is structurally related to tricyclic antidepressants, including CBZ (Table 1; Fig. 2). The phenothiazines have also been shown to enhance autophagic degradation of the aggregation-prone protein huntingtin (the protein mutated in Huntington’s disease) (Tsvetkov et al., 2010; Zhang et al., 2007). Thus, this type of screening platform provides a powerful new model for drug discovery for ATD and two new strategies for chemical and computational-based drug discovery using the autophagy enhancer drug paradigm and the phenothiazine structure.

In our initial study of CBZ, we speculated that a mechanism independent of TOR (target of rapamycin) kinase was involved in the autophagy-enhancing activity because we could not detect any effect of rapamycin on cellular ATZ load in a mammalian cell line or in the liver of the ATD mouse model (Hidvegi et al., 2010). Consistent with this, it seems that one of the phenothiazines discovered to enhance autophagic degradation of Huntington mediates its effects by a TOR-independent mechanism (Xia et al., 2010). In this case, autophagy was enhanced because of reduced intracellular calcium, which leads to diminished calpain-1-mediated cleavage of autophagy protein ATG5. We would predict that a number of different types of TOR-independent mechanisms for activation and enhancement of autophagy will be uncovered in the future, because more evidence for TOR-independent pathways for autophagy induction have recently emerged (Tan et al., 2012; Boglev et al., 2013).

**Table 1.**
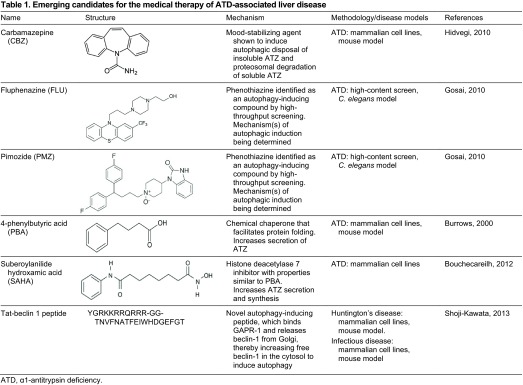
Emerging candidates for the medical therapy of ATD-associated liver disease.

Several other exciting strategies for enhancing autophagy have recently come to light. The laboratory of Beth Levine described a novel autophagy-inducing peptide termed Tat-beclin 1, building on prior observations that the HIV protein Nef inhibits autophagy by directly interacting with the autophagy regulatory factor beclin 1 (Kyei et al., 2009; Shoji-Kawata et al., 2013). In their experiments, Shoji-Kawata et al. succeeded in identifying an 18-amino acid Nef-interacting domain of beclin 1 and linked it to the Tat sequence to increase cell uptake (Shoji-Kawata et al., 2013). They went on to show that Tat-beclin 1 peptide is a potent inducer of autophagy and enhances the degradation of mutant huntingtin and several invasive bacterial and viral pathogens. Their findings suggest that Tat-beclin 1 could potentially be used as a therapeutic agent in ATD, in other diseases caused by aggregation-prone proteins and also possibly for certain infectious diseases.

Using a novel gene therapy approach, Pastore et al. studied the effect of transcription factor EB (TFEB), a master gene that regulates autophagy and lysosomal gene expression (Pastore et al., 2013). In experiments using mouse embryonic fibroblasts (MEFs) co-transfected with TFEB and ATZ, they found that TFEB induces ATZ clearance and that this effect was autophagy-dependent, because the reduction in ATZ load was not observed in autophagy-deficient *Atg7^−/−^* MEFs. Using adenovirus-mediated gene transfer of TFEB in the PiZ mouse model of ATD-associated liver disease, they found that TFEB transfer promoted hepatic ATZ clearance and reduced liver fibrosis in the mice. These findings further validate the concept that enhancing autophagy is a plausible therapeutic approach for ATD-associated liver disease (Table 1).

## Other new therapeutic strategies

Several other novel therapeutic approaches are emerging. In some cases these approaches are designed to target the gain-of-function proteotoxic mechanisms of ATD; however, several other of these new strategies target both gain-of-function and loss-of-function mechanisms to potentially ameliorate liver and lung disease. For example, two recent studies have suggested that gene therapy could be used to knockdown expression of the mutant *ATZ* gene using vectors that also encode the wild-type AT and therein would potentially address both gain-of-function proteotoxicity and loss-of-function mechanisms for tissue damage in ATD (Li et al., 2011; Mueller et al., 2012). In one study, adeno-associated virus encoding short-hairpin RNA to knockdown endogenous *AT* gene expression was used together with a codon-optimized wild-type *AT* transgene cassette (Li et al., 2011). In the other report, an adeno-associated virus encoding microRNA to silence endogenous *AT* gene expression together with a microRNA-resistant wild-type *AT* gene was delivered (Mueller et al., 2012). These approaches both led to high levels of human AT in the serum of a transgenic mouse model of ATD and significant reduction in hepatic ATZ accumulation. However, evidence that liver fibrosis was reduced by these strategies was not strong; thus, it remains uncertain whether or not more potent and widespread silencing would be needed within the liver to prevent proteotoxicity.

Several groups have utilized a strategy for ‘structure-based’ drug development that begins with designing peptides to prevent polymerization of the mutant ATZ molecule and theoretically influence its potential for secretion. For example, a small-molecule drug that targets a lateral hydrophobic cavity in the ATZ protein was found to prevent polymerization of ATZ but, in a cell model, it increased intracellular degradation rather than increased secretion (Mallya et al., 2007). Peptides that target the reactive center loop of AT have also been tested, and these increase the rate of secretion of mutant ATZ in a cell line model, although it is not clear whether they also led to increased intracellular accumulation (Alam et al., 2012). Furthermore, these peptides have not been tested in an animal model. Advancing the latter strategy will also depend on whether drugs based on the structure of these peptides can be identified and tested for safety and efficacy.

Chemical chaperones have also been investigated as a potential therapeutic class for treatment of ATD. These are compounds thought to optimize the intracellular folding environment in a general way and in so doing can at least partially correct cellular mislocalization of some mutant proteins (Engin and Hotamisligil, 2010). In contrast to so-called pharmacological chaperones, which bind to and stabilize proteins in a substrate-specific way, the chemical chaperones can facilitate folding of multiple misfolded proteins non-selectively. We have found that two of the compounds in the general category, glycerol and 4-phenylbutyric acid (PBA), mediate a marked increase in secretion of ATZ in a model cell line system (Burrows et al., 2000). This drug also mediated an increase in blood levels of human ATZ when administered orally to a mouse model transgenic for human *ATZ*, with blood levels reaching 20–50% of the levels present in normal humans. Because PBA had been used safely for years in children with urea-cycle disorders, this drug was considered a candidate for chemoprophylaxis of both liver and lung disease in ATD. Although a pilot study in ten patients with liver disease due to ATD did not show an increase in serum levels of AT after 14 days of treatment with PBA, it is possible that this was not a sufficient duration of treatment or that the ability of patients with chronic liver disease to tolerate the large dose of PBA required for effects in humans was limited (Teckman, 2004). Because PBA selectively affects secretion of ATZ and mediates its effect *in vivo*, and further because this kind of effect has not been observed for any other pharmacological agent, it will be important to further investigate PBA if more favorable formulations become available.

Recently a drug with chemical properties very similar to PBA, suberoylanilide hydroxamic acid (SAHA), was shown to increase secretion of ATZ in cell line models of ATD, and this effect was proposed to be mediated by inhibition of the histone deacetylase HDAC7 (Bouchecareilh et al., 2012). However, this drug was not tested *in vivo*. Furthermore, unlike PBA, SAHA mediates a substantial increase in synthesis of ATZ through a transcriptional activation mechanism. It was not entirely clear whether the increase in ATZ in extracellular fluid could be largely attributable to the increase in synthesis. Even if the results are consistent with increases in ATZ synthesis as well as an increase in translocation through the secretory pathway, the effect of this drug would be associated with increased accumulation of ATZ in the ER and therein possibly with increased proteotoxicity (Table 1).

Because transplanted hepatocytes can repopulate the diseased liver, cell transplantation therapy for ATD has also been discussed. Indeed, Ding et al. demonstrated that wild-type donor hepatocytes can almost completely repopulate the liver of the PiZ mouse model of ATD (Ding et al., 2011). Use of this type of ‘cellular’ therapy could be an alternative approach to replacement therapy to prevent COPD and perhaps also to prevent or treat liver disease, because the transplanted hepatocytes have a selective proliferative advantage over endogenous hepatocytes and can eventually supplant the latter.

A combination of gene-targeting and cell-based therapy might also be a potential strategy for COPD and perhaps liver disease in ATD. Yusa et al. recently reported exciting results in which the mutation in the *AT* gene was corrected in human induced pluripotent stem cells (iPS cells) from a patient homozygous for *ATZ* by a combination of zinc-finger nucleases and transposon technology, and then the iPS cell line was shown to engraft into the liver of a transgenic mouse model (Yusa et al., 2011). This strategy, if it can be adapted for human application, has the potential to correct both the loss-of-function and gain-of-function mechanisms, and would have the advantage of obviating the need for immunosuppression.

## Conclusions and future directions

ATD is unique in that the disease phenotype in one target organ, the lung, is caused by a loss-of-function mechanism, whereas, in another organ, the liver, it is caused by gain-of-function proteotoxicity. ATD is the most common genetic cause of COPD, but modifiers such as cigarette smoking and consequences of infections play an important role in varying the age of onset and severity of lung disease. Although replacement therapy with purified AT corrects the loss-of-function mechanism for lung disease in ATD, this therapy has not benefited all affected individuals and has had limited clinical efficacy in other cases.

ATD is also the most common genetic cause of liver disease in childhood. Different clinical forms present in infancy, childhood, adolescence or during adulthood. Epidemiological information suggests that genetic and/or environmental modifiers determine whether or not ATD individuals develop clinically significant liver disease and its age of onset. A series of studies suggest that endogenous proteostasis mechanisms are candidates for these modifiers and autophagy is a particularly important mechanism by which the liver attempts to protect itself from proteotoxicity. Exciting new data indicates that FDA-approved drugs that enhance autophagy reduce liver disease in mouse models of ATD and could be ideal candidates for chemoprophylaxis of ATD-associated liver disease. Several other new therapeutic strategies, including gene silencing and the use of genetically engineered iPS-cell-derived hepatocytes, are now under investigation.

The major challenges for clinical and basic research in the near-term include further elucidation of modifiers and characterization of how these modifiers act in cell and animal models (see Clinical and basic research opportunities box). The use of iPS cell line models will help to understand the action of modifiers on ATZ accumulation/proteotoxicity and also to provide a personalized approach to the testing of treatment strategies. Further studies of why AT replacement therapy has not had a more transformational effect on lung disease progression and of the prevalence of liver disease among adults with ATD are needed. Identification of biomarkers of the hepatic and pulmonary diseases of ATD will be essential for clinical trials of the new therapeutic strategies currently being developed.

**Clinical and basic research opportunities**

- Prevalence of liver and lung disease in adults with ATD
- Identification of modifiers of ATD hepatic disease
- Further elucidation of modifiers of ATD lung disease Development of iPS cell line models to determine the mechanism of action of modifiers and for personalized testing of novel therapeutic strategies
- Screening of drug and RNAi libraries using the *C. elegans* model of ATD and computational pharmacological techniques for drug discovery
- Identification of biomarkers and design strategies for clinical trials of novel therapies
- Further studies of why AT replacement therapy does not have a more dramatic effect on the progression of lung disease due to ATD
- Further studies of the pathobiology of hepatocellular carcinoma in ATD.
